# Geriatric Nutritional Risk Index (GNRI) and Creatinine Index Equally Predict the Risk of Mortality in Hemodialysis Patients: J-DOPPS

**DOI:** 10.1038/s41598-020-62720-6

**Published:** 2020-04-01

**Authors:** Shunsuke Yamada, Shungo Yamamoto, Shingo Fukuma, Toshiaki Nakano, Kazuhiko Tsuruya, Masaaki Inaba

**Affiliations:** 1 Department of Medicine and Clinical Science, Graduate School of Medical Sciences, Kyushu University, Fukuoka, Japan; 2 Department of Healthcare Epidemiology, School of Public Health in the Graduate School of Medicine, Kyoto University, Kyoto, Japan; 3 Institute for Health Outcomes and Process Evaluation Research (iHope International), Kyoto, Japan; 4 Human Health Sciences, Graduate School of Medicine, Kyoto University, Kyoto, Japan; 5 Department of Nephrology, Nara Medical University, Nara, Japan; 6 Department of Metabolism, Endocrinology and Molecular Medicine, Osaka City University Graduate School of Medicine, Osaka, Japan

**Keywords:** End-stage renal disease, Haemodialysis

## Abstract

The geriatric nutritional risk index (GNRI) and creatinine (Cr) index are indexes often used as nutritional surrogates in patients receiving hemodialysis. However, few studies have directly compared the clinical characteristics of these two indexes. We investigated 3,536 hemodialysis patients enrolled in the Japan DOPPS phases 4 and 5. The primary outcome was all-cause mortality and the main exposures were the GNRI and Cr index. We confirmed and compared the association between these indexes and mortality risk as estimated by a multivariable-adjusted Cox proportional hazards model. During the median 2.2-year follow-up period, 414 patients died of any cause. In the multivariable-adjusted model, lower GNRI and Cr index were both associated with increased risk of all-cause mortality, and these associations were further confirmed by restricted cubic spline curves. The predictability of all-cause mortality, as represented by the c-statistic, was comparable between the two indexes. Furthermore, baseline nutritional surrogates that corresponded with lower GNRI or Cr index values were comparable between the two indexes. Given that calculating the GNRI is simpler than calculating the Cr index, our data suggest that the GNRI may be preferable to the Cr index for predicting clinical outcomes in patients undergoing maintenance hemodialysis.

## Introduction

Malnutrition is highly prevalent in patients receiving maintenance hemodialysis^1–3^. Inflammation often coexists with malnutrition. Because these two pathologies synergistically promote clinically important complications, including atherosclerotic diseases, they are now jointly recognized by the integrated term “malnutrition-inflammation-atherosclerosis (MIA) syndrome” or “malnutrition-inflammation complex/cachexia syndrome (MICS)”^4,5^. Identification of objective markers that both reflect MIA syndrome and can be used for daily evaluation of nutritional and inflammatory status in this population is now urgently required.

A wide variety of nutritional and inflammatory markers and tools have been reported for the evaluation of MIA syndrome or MICS in hemodialysis patients. Because these markers and indexes are insufficient when used alone, many clinicians use them in combination in clinical practice. These include subjective global assessment (SGA); malnutrition-inflammation score (MIS); serum levels of albumin, creatinine (Cr), and C-reactive protein (CRP); body mass index (BMI); normalized protein catabolic rate (nPCR); interleukin-6; geriatric nutritional risk index (GNRI); Cr index; Objective Score of Nutrition on Dialysis (OSND); simple protein energy wasting score; Subjective Global Assessment-Dialysis Malnutrition Score (SGA-DMS); bioelectrical impedance analysis (BIA); and dual energy X-ray absorptiometry (DEXA)^3,5–15^. Of these, the GNRI and Cr index are often used to evaluate nutritional status in hemodialysis patients^9,10^. The GNRI is calculated by serum albumin level and BMI, while the Cr index is determined by age, gender, Kt/V for urea, and pre-dialysis serum Cr level. These two indexes are objective and do not require special techniques or experience but are instead easily calculated by routine blood test results obtained at the bedside. Furthermore, both have been shown to be associated with increased risk for mortality^16–18^. For GNRI, meta-analyses have confirmed its usefulness for good predictability of mortality^19,20^. Although these indexes reflect nutritional status, they are derived from different clinical parameters and are thus unlikely to impact clinically relevant outcomes in the same way in patients receiving maintenance hemodialysis. In addition, because the GNRI only requires two parameters whereas the Cr index requires four, calculating the GNRI is simpler. If outcome predictability is comparable between these two indexes, the simpler of the two would be more practical and useful in the clinical setting. Despite these, few studies have compared the usefulness and predictive ability of these two nutritional indexes regarding all-cause and cardiovascular mortality in this population^21^. It is clinically important to directly compare these two indexes in the same study population comprising a relatively large number of hemodialysis patients.

There were two main aims of the present study. One was to confirm the previously established observations that lower GNRI and Cr index values are associated with increased risk of all-cause death in maintenance hemodialysis patients. The second was to determine whether the GNRI and the Cr index are equally valuable, or whether one is preferable to the other as a surrogate of nutritional status and mortality in hemodialysis patients.

## Results

### Patient characteristics

In the present study, we used the dataset of phases 4 and 5 of the Japan Dialysis Outcomes and Practice Patterns Study (DOPPS), a multicenter, prospective, observational study conducted in Japan as part of the DOPPS^22,23^. Japan DOPPS phases 4 and 5 included a total of 4,806 participants. After exclusion of participants with missing baseline GNRI (n = 675) or Cr index (n = 905) values, 3,536 patients were deemed eligible for the present study. Baseline characteristics stratified by GNRI and Cr index quartiles are shown in Table 1. Median age was 66 (58–74) years and 65.0% were male. Median dialysis vintage was 4.2 (0.8–10.1) years. When divided into quartiles based on GNRI, patients with a lower GNRI were older and showed a higher prevalence of female, higher prevalence of a history of cardiovascular diseases, lower body weight and BMI, lower systolic blood pressure level, higher single pool Kt/V for urea, lower serum levels of albumin, Cr, urea nitrogen, calcium, phosphate, parathyroid hormone (PTH), higher serum level of alkaline phosphatase, and lower prescription proportion of vitamin D receptor activators (VDRAs), phosphate binders, and erythropoiesis-stimulating agents. When divided into quartiles based on Cr index, patients with a lower Cr index were older and showed a higher prevalence of female, shorter dialysis vintage, higher prevalence of diabetes mellitus and history of cardiovascular diseases, lower body weight and BMI, higher prevalence of presence of residual kidney function (RKF), lower systolic blood pressure level, lower serum levels of albumin, Cr, urea nitrogen, calcium, phosphate, PTH, higher serum level of alkaline phosphatase, and lower prescription proportion of VDRAs, phosphate binders, and erythropoiesis-stimulating agents.

**Table 1:** Baseline patient characteristics stratified by quartiles of GNRI and Cr index.

Baseline characteristics	Overall (n = 3536)	Quartiles of GNRI (n = 3536)					Quartiles of Cr index (n = 3536)				
		Q1 (≤89.8) (n = 891)	Q2 (89.9 to 95.2) (n = 879)	Q3 (95.3 to 99.0) (n = 890)	Q4 (≥99.1) (n = 876)	*P* for trend	Q1 (≤18.9) (n = 889)	Q2 (19.0 to 20.8) (n = 892)	Q3 (20.9 to 22.9) (n = 883)	Q4 (≥23.0) (n = 872)	*P* for trend
Age, years	66 (58–74)	70 (63–78)	67 (61–75)	65 (57–72)	61 (52–69)	<0.001	74 (67–80)	68 (62–75)	64 (59–71)	56 (47–63)	<0.001
Gender, male, %	65.0	58.5	62.6	66.4	72.6	<0.001	41.7	56.5	73.4	88.9	<0.001
Dialysis vintage, years	4.2 (0.8–10.1)	3.7 (0.4–10.8)	4.9 (0.9–12.0)	4.3 (1.0–9.6)	3.9 (1.1–8.7)	0.16	1.2 (0.3–5.2)	3.6 (0.6–9.0)	5.8 (2.9–12.1)	6.7 (2.9–12.8)	<0.001
Body weight, kg	56.5 (48.8–64.3)	49.0 (43.0–56.4)	54.2 (47.5–60.9)	59.5 (52.9–67.1)	62.4 (55.3–69.3)	<0.001	50.2 (44.0–57.6)	53.8 (47.0–61.1)	57.8 (50.8–65.1)	63.4 (56.5–70.1)	<0.001
Diabetes mellitus, %	39.9	39.4	39.0	39.7	41.6	0.34	49.6	45.1	37.7	27.0	<0.001
CVDs, %	56.3	61.5	61.1	53.3	49.2	<0.001	67.0	59.1	54.1	44.6	<0.001
SBP, mmHg	150 (134–165)	145 (130–162)	148 (134–165)	150 (134–165)	151 (136–167)	<0.001	147 (130–163)	149 (134–165)	150 (134–164)	150 (136–167)	0.002
Single pool Kt/V	1.3 (1.2–1.5)	1.4 (1.1–1.6)	1.4 (1.2–1.6)	1.3 (1.2–1.5)	1.3 (1.1–1.5)	<0.001	1.3 (1.1–1.6)	1.4 (1.2–1.6)	1.4 (1.2–1.6)	1.3 (1.2–1.5)	0.31
nPCR, g/kg/day	0.9 (0.8–1.1)	0.9 (0.7–1.0)	0.9 (0.8–1.1)	0.9 (0.8–1.1)	1.0 (0.8–1.1)	<0.001	0.8 (0.7–1.0)	0.9 (0.8–1.0)	1.0 (0.8–1.1)	1.0 (0.9–1.1)	<0.001
BMI, kg/m^2^	21.1 (19.0–23.4)	18.7 (17.1–20.6)	20.1 (18.7–22.4)	22.2 (20.4–24.2)	22.4 (21.0–24.4)	<0.001	20.2 (18.1–22.7)	20.6 (18.6–23.1)	21.1 (19.3–23.4)	22.0 (20.2–24.0)	<0.001
Presence of RKF, %	12.1	11.2	11.7	13.9	11.6	0.47	17.3	13.2	9.9	8.0	<0.001
Serum albumin, g/dL	3.7 (3.4–3.9)	3.2 (3.0–3.4)	3.6 (3.5–3.8)	3.8 (3.7–3.8)	4.1 (4.0–4.2)	<0.001	3.5 (3.2–3.8)	3.7 (3.4–3.9)	3.7 (3.5–3.9)	3.9 (3.7–4.1)	<0.001
Serum CRP, mg/dL	0.12 (0.06–0.40)	0.25 (0.10–1.02)	0.12 (0.06–0.30)	0.10 (0.05–0.28)	0.10 (0.05–0.26)	<0.001	0.20 (0.08–0.60)	0.13 (0.06–0.40)	0.10 (0.06–0.30)	0.10 (0.05–0.30)	<0.001
BUN, mg/dL	62.8 (52.4–73.5)	58.4 (46.0–70.0)	61.0 (51.8–72.4)	64.0 (55.0–73.6)	66.7 (57.7–77.2)	<0.001	54.5 (44.6–66.5)	60.0 (50.7–70.0)	65.0 (56.0–75.0)	70.0 (61.0–79.2)	<0.001
Serum Cr, mg/dL	10.0 (8.1–12.1)	8.4 (6.6–10.2)	9.8 (8.2–11.5)	10.7 (8.8–12.6)	11.3 (9.3–13.6)	<0.001	6.7 (5.6–7.7)	9.1 (8.4–9.8)	11.0 (10.3–11.6)	13.7 (12.8–14.8)	<0.001
Serum calcium, mg/dL	8.8 (8.3–9.3)	8.5 (8.0–8.9)	8.8 (8.3–9.3)	8.9 (8.4–9.4)	9.1 (8.6–9.5)	<0.001	8.6 (8.1–9.1)	8.8 (8.3–9.3)	8.9 (8.4–9.4)	9.0 (8.6–9.5)	<0.001
Serum Pi, mg/dL	5.3 (4.5–6.2)	4.9 (4.1–5.9)	5.1 (4.4–5.9)	5.5 (4.7–6.2)	5.7 (4.9–6.6)	<0.001	4.8 (4.0–5.5)	5.1 (4.4–6.0)	5.5 (4.8–6.3)	5.8 (5.0–6.7)	<0.001
Serum ALP, U/L	234 (186–309)	249 (198–324)	247 (196–322)	223 (176–296)	222 (181–287)	<0.001	258 (202–331)	244 (194–328)	231 (181–307)	219 (176–279)	<0.001
Serum PTH, pg/mL	127 (66–210)	114 (52–180)	138 (72–214)	129 (73–212)	130 (69–218)	0.03	123 (65–187)	122 (60–199)	127 (67–220)	135 (71–224)	0.07
Use of VDRAs, %	48.2	38.2	50.1	51.1	53.5	<0.001	35.1	45.2	54.3	58.5	<0.001
Use of Pi-binders, %	57.8	45.0	61.1	59.9	66.2	<0.001	35.2	52.5	68.7	75.1	<0.001
Use of ESAs, %	58.7	55.2	60.3	56.7	62.6	0.01	50.4	58.7	66.1	59.5	<0.001

Data are expressed as median (95% CI) or percentage. Abbreviations: ALP, alkaline phosphatase; BMI, body mass index; BUN, Blood urea nitrogen; CI, confidence interval; Cr, creatinine; CRP, C-reactive protein; CVDs, cardiovascular diseases, ESAs, erythropoiesis-stimulating agents; GNRI, geriatric nutritional risk index; nPCR, normalized protein catabolic rate; Pi, phosphate; PTH, parathyroid hormone; RKF, residual kidney function; SBP, systolic blood pressure; VDRAs, vitamin D receptor activators.

### Kaplan-Meier curves for all-cause and cardiovascular death stratified by GNRI or Cr index

**Figure 1:**
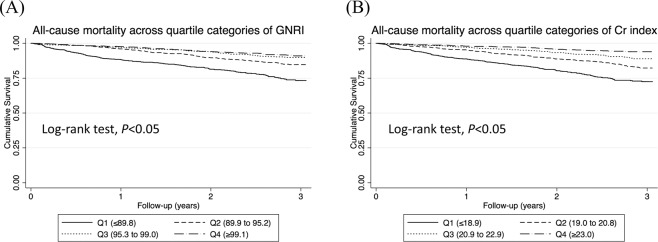
Kaplan-Meier curves for all-cause mortality stratified by the two nutritional indexes. (**A**) GNRI quartile and (**B**) Cr index quartile. The log-rank test was used in analysis. A two-tailed *P* < 0.05 was considered statistically significant. Abbreviations: Cr, creatinine; GNRI, geriatric nutritional risk index.

During the median observation period of 2.2 years, 414 patients died of any cause and 151 patients died of cardiovascular diseases. Figure 1 shows non-adjusted Kaplan-Meier curves for all-cause death according to groups stratified by GNRI or Cr index. Patients in a lower GNRI category showed a significantly higher incidence rate of all-cause death than those in a higher GNRI category (Log-rank test, *P* < 0.05). Similarly, patients in a lower Cr index category showed a significantly higher incidence rate of all-cause death than patients in a higher Cr index category (Log-rank test, *P* < 0.05).

Regarding cardiovascular deaths, patients in a lower GNRI category showed a significantly higher cardiovascular mortality than patients with a higher GNRI category (Fig. S1A). Similarly, patients in a lower Cr index category showed a significantly higher cardiovascular mortality than patients in a higher Cr index category (Fig. S1B).

### Association between the risk for all-cause and cardiovascular death and two nutritional indexes examined by cox proportional hazards models

To determine the association between the two nutritional indexes and death, we estimated the hazards risks for all-cause and cardiovascular deaths in each quartile by applying Cox proportional hazards risk models. As shown in Table 2, an age- and gender-adjusted Cox proportional hazards model showed that a lower GNRI category was significantly associated with an increased risk for all-cause death compared with the highest GNRI category. The association remained unchanged even after adjustment for potential confounding factors: multivariable-adjusted hazard risk (HR) [95% confidence interval (CI)] of Q1 was 2.21 [1.61–3.03] compared with the reference group (Q4). As for cardiovascular mortality, the association between GNRI and cardiovascular mortality was only marginally significant (Table 2).

**Table 2:** Adjusted HRs (95% CI) of all-cause and cardiovascular mortality across quartiles of GNRI.

	Harrell’s c-index	Quartiles of GNRI				*P* for trend
		Q1 (≤89.3) (n = 1080)	Q2 (89.4 to 94.9) (n = 981)	Q3 (95.0 to 98.7) (n = 1029)	Q4 (≥98.8) (n = 1015)	
**All-cause mortality**						
Model 1	0.726 (0.701, 0.750)	2.24 (1.60, 3.13)	1.27 (0.91, 1.78)	0.94 (0.66, 1.34)	1 [reference]	<0.001
Model 2	0.749 (0.725, 0.772)	2.21 (1.61, 3.03)	1.19 (0.85, 1.66)	0.95 (0.67, 1.34)	1 [reference]	<0.001
**Cardiovascular mortality**						
Model 1	0.708 (0.668, 0.749)	1.60 (0.95, 2.70)	0.93 (0.52, 1.64)	1.20 (0.72, 1.98)	1 [reference]	0.19
Model 2	0.747 (0.710, 0.783)	1.61 (0.98, 2.62)	0.87 (0.50, 1.54)	1.20 (0.73, 1.98)	1 [reference]	0.19

Data are expressed as HR (95% CI).

Model 1 was adjusted for age and gender.

Model 2 was adjusted for age, gender, dialysis vintage, and comorbidity (history of diabetes mellitus and cardiovascular diseases).

A *P*-value < 0.05 was considered statistically significant.

Abbreviations: CI, confidence interval; GNRI, geriatric nutritional risk index; HR, hazards ratio; Q, quartile.

The age- and gender-adjusted Cox proportional hazards model showed that the lowest Cr index category was significantly associated with an increased risk for all-cause death compared with the highest Cr index category (Table 3). This association remained unchanged even after adjustment for potential confounding factors: the multivariable-adjusted HR [95% CI] of Q1 was 3.49 [2.08–5.85] compared with the reference group (Q4). As for cardiovascular mortality, the association between Cr index and cardiovascular mortality was also statistically significant (Table 3), with a multivariable-adjusted HR [95% CI] of Q1 of 3.07 [1.14–8.22] compared with the reference group (Q4)(Model 2).

**Table 3:** Adjusted HRs (95% CI) of all-cause and cardiovascular mortality across quartiles of Cr index.

	Harrell’s c-index	Quartiles of Cr index				*P* for trend
		Q1 (≤18.9) (n = 969)	Q2 (19.0 to 20.9) (n = 1021)	Q3 (21.0 to 23.0) (n = 955)	Q4 (≥23.1) (n = 930)	
**All-cause mortality**						
Model 1	0.729 (0.704, 0.753)	3.32 (2.08, 5.29)	2.00 (1.31, 3.06)	1.28 (0.83, 1.99)	1 [reference]	<0.001
Model 2	0.751 (0.728, 0.775)	3.49 (2.08, 5.85)	1.96 (1.24, 3.11)	1.27 (0.81, 1.98)	1 [reference]	<0.001
Model 3	0.752 (0.729, 0.775)	3.58 (2.14, 5.98)	2.00 (1.26, 3.15)	1.28 (0.82, 2.00)	1 [reference]	<0.001
**Cardiovascular mortality**						
Model 1	0.723 (0.685, 0.762)	3.26 (1.37, 7.74)	2.11 (0.95, 4.70)	1.51 (0.69, 3.30)	1 [reference]	0.005
Model 2	0.754 (0.719, 0.789)	3.07 (1.14, 8.22)	1.95 (0.80, 4.78)	1.43 (0.62, 3.29)	1 [reference]	0.02
Model 3	0.755 (0.721, 0.790)	3.15 (1.18, 8.42)	1.99 (0.82, 4.84)	1.45 (0.63. 3.32)	1 [reference]	0.02

Data are expressed as HR (95% CI).

Model 1 was adjusted for age and gender.

Model 2 was adjusted for age, gender, dialysis vintage, and comorbidity (diabetes mellitus and cardiovascular diseases).

Model 3 was adjusted for age, gender, dialysis vintage, comorbidity (history of diabetes mellitus and cardiovascular diseases), and presence of RKF. Presence of RKF was defined as presence of daily urine volume > 200 mL/day.

A *P*-value < 0.05 was considered statistically significant.

Abbreviations: CI, confidence interval; Cr, creatinine; HR, hazards ratio; Q, quartile; RKF, residual kidney function.

To further address the impact of RKF on Cr index, we conducted a sensitivity analysis. As shown in Table 3, even when RKF was included as a covariate in the multivariable analysis, the association remained almost unchanged (Model 3).

Finally, when we compared c-statistics between the GNRI and Cr index regarding all-cause and cardiovascular mortality, no significant differences were observed, as shown in Tables 2 and 3.

### Non-linear dose-response associations between risk for all-cause and cardiovascular death and two nutritional indexes by restricted cubic spline regression

**Figure 2:**
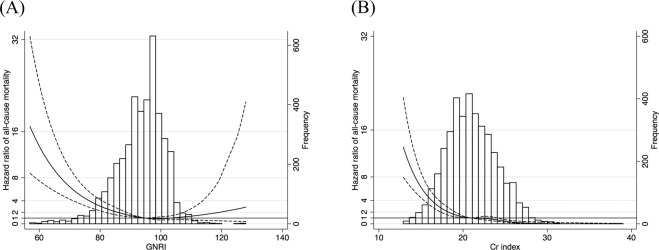
Multivariable-adjusted restricted cubic spline plots of HR for all-cause mortality according to the two nutritional indexes. (**A**) GNRI quartile and (**B**) Cr index quartile. Solid line represents HR and dotted line represents 95% confidence interval. The horizontal line corresponds to the normal reference HR of 1.0. The overall median value of GNRI and Cr index were 95.2 and 20.8 mg/kg/day, respectively, and were chosen as the references. The multivariable-adjusted model was adjusted for age, gender, dialysis vintage, and comorbidity (diabetes mellitus and cardiovascular diseases). A two-tailed *P* value of < 0.05 was considered to indicate statistical significance. Abbreviations: Cr, creatinine; GNRI, geriatric nutritional risk index; HR, hazard ratio.

We also determined non-linear associations between nutritional indexes and all-cause mortality by multivariable-adjusted Cox proportional hazards models with restricted cubic spline regression. As shown in Fig. 2A, the adjusted hazard ratio for all-cause mortality was significantly increased when the GNRI was lower than 96 compared with those patients with GNRI ≥ 96, showing a non-linear association between GNRI and mortality risk. By contrast, patients with a Cr index of <21 mg/kg/day showed an increased risk for all-cause mortality, while those with a Cr index > 24 mg/kg/day showed a decreased risk compared with those with a Cr index between 21 and 24 mg/kg/day, showing an almost linear association (Fig. 2B). Overall, the Cr index showed a decremental association with all-cause mortality across a wide range of Cr index values. The associations between these two indexes and all-cause mortality remained almost unchanged even when the following baseline factors were further added to the multivariable analysis after imputation of missing data by mean values: systolic blood pressure level, normalized protein catabolic rate, Kt/V for urea, blood hemoglobin, serum levels of CRP, calcium, phosphate, and PTH (Fig. S2A,B).

As for cardiovascular death, the GNRI showed a negative association with cardiovascular mortality risk with a GNRI value of 91.5 as the lowest (Fig. S3A). In contrast, the Cr index showed a decremental association with cardiovascular mortality across a wide range of Cr index values (Fig. S3B). The associations between these two indexes and cardiovascular mortality remained almost unchanged even when the following baseline factors were further added to the multivariable analysis after imputation of missing data by mean values: systolic blood pressure level, normalized protein catabolic rate, Kt/V for urea, blood hemoglobin, serum levels of CRP, calcium, phosphate, and PTH (Fig. S4A,B).

### Comparison of factors determining the presence of malnutrition defined by a lower GNRI or lower Cr index

To clarify potentially differential characteristics of the GNRI and Cr index as indicators of nutritional status, we also compared those factors determining malnutrition as defined by a low GNRI or a low Cr index using multivariable-adjusted logistic regression analyses. As shown in Table 4, malnutrition determined by either a low GNRI or a low Cr index was consistently associated with lower nPCR and lower BMI. There was a significant association between serum Cr and malnutrition determined via a low GNRI, and there was a significant association between serum albumin and malnutrition determined via a low Cr index. Serum C-reactive protein was marginally associated with malnutrition determined by a low GNRI

**Table 4:** Comparison of variables associated with “malnutrition” defined by a low GNRI category or a low Cr index category.

Variables	Adjusted odds ratio (95% CI) for a low GNRI category	*P*-value	Adjusted odds ratio (95% CI) for a low Cr index category	*P*-value
Normalized protein catabolic rate, per 1 g/kg/day increase	0.34 (0.19, 0.63)	0.001	0.05 (0.02, 0.10)	<0.001
Body mass index, per 1 kg/m^2^ increase	0.71 (0.68, 0.75)	<0.001	0.91 (0.87, 0.94)	<0.001
Serum Cr, per 1 mg/dL increase	0.83 (0.78, 0.87)	<0.001	—	—
Serum albumin, per 1 g/dL increase	—	—	0.42 (0.31, 0.58)	<0.001
Serum CRP, per 1 mg/dL increase	1.00 (1.00, 1.01)	0.07	1.00 (1.00, 1.00)	0.99

A low GNRI was defined as the patients in the lowest quartile (Q1) of GNRI. A low Cr index was defined as the patients in the lowest quartile (Q1) of Cr index. AUCs calculated by multivariable adjusted logistic regression analysis for GNRI and Cr index were 0.814 (0.793 to 0.835) and 0.900 (0.885 to 0.913), respectively. Age, sex, dialysis vintage, history of diabetes mellitus and cardiovascular disease, and single-pool Kt/V for urea, use of vitamin D receptor activators and phosphate binders were also included in the multivariable-adjusted logistic regression analysis. A *P*-value less than 0.05 was considered statistically significant. Abbreviations: AUC, area under the curve; CI, confidence interval; Cr, creatinine; CRP, C-reactive protein; GNRI, geriatric nutritional risk index.

## Discussion

In this study, we confirmed that lower GNRI and Cr index were associated with almost equal increases in risk for all-cause mortality in maintenance hemodialysis patients, consistent with the observations obtained by the restricted cubic spline curves. Regarding model predictability, no significant difference was observed between GNRI and Cr index for the c-statistic of all-cause mortality. Furthermore, the baseline clinical factors associated with malnutrition defined by lower GNRI or Cr index were comparable between the two indexes. These results suggest that the GNRI, a simpler surrogate of nutritional status, may be preferable to the Cr index for predicting mortality risk in hemodialysis patients. The results of the present study are summarized in Supplementary Table 1.

In our present study, patients in lower GNRI and Cr index categories equally showed a significantly increased risk for all-cause mortality. Although the GNRI and Cr index are calculated using a different set of clinical parameters, our results suggest that they partially share nutritional information regarding the predictability of all-cause mortality. One very recent study compared the model predictability of the GNRI and Cr index regarding all-cause mortality in a relatively small cohort of 88 Chinese hemodialysis patients^21^. Results showed that model performance for all-cause mortality with the Cr index was better than that with the GNRI. Although we have no clear explanation for this discrepancy between the present study and this previous report, one possibility is the difference in the baseline nutritional status of the two cohorts. A second possibility is the relatively short observation period of our study. In any case, our present findings should be confirmed in other hemodialysis cohorts comprised of diverse ethnic groups.

As shown in Table 4, baseline parameters related to nutritional status were shared between the GNRI and the Cr index with regard to malnutrition as determined by a low GNRI or a low Cr index. These parameters included nPCR and BMI. Both BMI and nPCR were reliable markers for malnutrition in hemodialysis patients, and our observation therefore appeared reasonable. Malnutrition defined by low GNRI was associated with lower serum Cr, whereas malnutrition defined by a low Cr index was associated with lower serum albumin. In a recent study infection-related and all-cause death predicted by the GNRI and by serum Cr were comparable, indicating that serum albumin-based surrogates and serum Cr-based surrogates are equally useful for the prediction of mortality in hemodialysis patients^24^. Both GNRI and Cr index are found to be valid tools for longitudinal observation of nutritional status in patients receiving maintenance hemodialysis^21,25^. Given that outcome predictability and nutritional information derived from the GNRI and the Cr index are comparable, the GNRI—a simpler form of nutritional surrogate—may be more useful and practical for clinical use than the Cr index in patients undergoing maintenance hemodialysis.

The question of whether these two indexes accurately predict other outcomes apart from all-cause and cardiovascular mortality in hemodialysis patients remains unanswered. We very recently showed that a lower Cr index was associated with an increased risk for bone fracture^26^. To date, however, no studies have focused on the association between GNRI and bone fracture. Because previous studies focused on cardiovascular and all-cause mortality, further studies examining the impact of these two indexes on a variety of clinically important outcomes in hemodialysis patients are necessary. It is probable that the outcome predictability may differ between the two indexes depending on the types of outcomes examined. These should finally indicate which index is clinically more useful as a nutritional index that can differentiate risk for important outcomes in hemodialysis patients or it is possible that we should differentially use these two indexed depending on the outcomes. Hence, further studies that focus on other clinical outcomes are necessary to compare the usefulness of the two indexes.

The major strengths of this study are its population-based design, large sample size, replication cohort, and use of standardized data across facilities^22,23,27^. Participants in the DOPPS are representative dialysis patients of particular countries selected via a stratified random sampling method^28^. We are also aware of several limitations of our study. First, both the GNRI and Cr index were obtained only at baseline. Since both fluctuate with changes in the patients’ medical condition, changes in these nutritional indexes may affect the association between them and outcomes. Second, given the nature of observational studies, causal relationships cannot be examined. Third, unmeasured and residual confoundings might have affected the results of our study. Fourth, we did not compare the model performance of these nutritional indexes with MIS, one of the standard nutritional assessment tools frequently used in hemodialysis patients^5^. Fifth, the present study included hemodialysis patients with RKF. The Cr index was originally developed for patients without RKF. When Cr index is applied to patients with RKF, total daily Cr excretion may be underestimated. However, in the present study, Cr index showed significant associations with all-cause and cardiovascular mortality, even after adjustment for RKF. These results suggest that Cr index can be used as a surrogate for mortality, at least when applied to a relatively large study population in which the proportion of patients with RKF is relatively minor compared with that of patients without RKF. Allowing for these limitations, we believe that our results provide useful information about the GNRI and Cr index as nutritional surrogates in hemodialysis patients and serve to direct future goals in this field.

In conclusion, this study confirmed that lower GNRI and Cr index values were almost equally associated with increased risk for all-cause mortality in hemodialysis patients. Because the baseline values that determined a lower GNRI or Cr index category were almost comparable, the GNRI may be more useful than the Cr index for the prediction of mortality in hemodialysis patients because it is a simpler surrogate of nutritional status. Further studies are necessary to determine whether there are differential associations between these two nutritional indexes and clinically important outcomes other than all-cause and cardiovascular deaths in maintenance hemodialysis, including bone fracture, infection-related death, and hospitalization.

## Materials and Methods

### Design, setting and participants

We used data from phases 4 (2009–2011) and 5 (2012–2014) of the Japan DOPPS (J-DOPPS), which is part of the DOPPS, an international prospective cohort study of in-center hemodialysis patients. The design, data elements, and methodology of the DOPPS have been detailed elsewhere^22,23^. Participants in the J-DOPPS were randomly selected from a nationally representative sample of dialysis facilities in Japan^23^ and provided written informed consent for inclusion in the study. To be eligible, participants were required to have no missing baseline data for the GNRI or Cr index. The study was performed according to the Ethics of Clinical Research (Declaration of Helsinki). This study’s conduct was approved by a central ethics committee (Tokyo Women’s Medical University, approval numbers 1527 and 2388 for phases 4 and 5, respectively).

### Calculation of the nutritional indexes

The two nutritional indexes were each calculated from the baseline data. The GNRI was calculated using the following formula^9^:

GNRI = [14.89 × serum albumin (g/dL)] + [41.7 × (actual body weight/ideal body weight)],

where ideal body weight was calculated as follows:

ideal body weight (kg) = [height (m)]^2^ × 22 (kg/m^2^).

We set (actual body weight/ideal body weight) as 1 when a patient’s actual body weight was equal to or over the ideal body weight.

Cr index was calculated as follows based on known sex differences^10,18^:

Cr index for men = 16.21 + 1.12–0.06 × [age (year)] − 0.08 ×(single pool Kt/V) + 0.009 ×[serum creatinine (μmol/L)],

Cr index for women = 16.21–0.06 ×[age (year)] − 0.08 × (single pool Kt/V) + 0.009 × [serum creatinine (μmol/L)].

GNRI and Cr index results were divided into quartiles, and each patient was then assigned to one of four categories for each.

### Outcomes

The primary outcome of interest was the incidence of all-cause death. The date and cause of death in the J-DOPPS were determined roughly every 4 months. Participants were followed until death or other censoring events, including loss to follow-up, or the end of follow-up for this study. Incidence of death due to cardiovascular causes was treated as the secondary outcome. Cardiovascular death included death due to acute myocardial infarction, pericarditis (including cardiac tamponade), atherosclerotic heart disease, cardiomyopathy, cardiac arrhythmia, cardiac arrest due to unknown cause, valvular heart disease, pulmonary edema due to exogenous fluid, congestive heart failure, pulmonary embolus, cerebrovascular accident, including intracranial hemorrhage, ischemic brain damage, hemorrhage from a transplant site, vascular access, dialysis circuit, ruptured vascular aneurysm, surgery, or other hemorrhage, mesenteric infarction/ischemic bowel, or calciphylaxis.

### Statistical analysis

Baseline patient characteristics were summarized according to categories of GNRI and Cr index, respectively, and presented as percentages for categorical variables and medians (interquartile ranges) for continuous variables. We calculated *P* for trend in each variable across the four categories using Cuzick’s nonparametric test for trend^29^.

First, we estimated survival using the Kaplan-Meier method, and compared differences using the log-rank test. Second, we used restricted cubic splines to determine the association of mortality with the GNRI and Cr index, adjusted for age, gender, dialysis vintage, and comorbidity (diabetes mellitus and cardiovascular diseases)^30^. As a sensitivity analysis, we further included systolic blood pressure level, normalized protein catabolic rate, Kt/V for urea, blood hemoglobin, and serum levels of CRP, calcium, phosphate, and PTH as covariates in the restricted cubic spline analyses after imputation of mean values, because some of these parameters were missing in a few patients. Third, we assessed the associations of the GNRI and Cr index with outcomes using Cox proportional hazards models, accounting for facility clustering using robust sandwich covariance estimators^22^. Model 1 was adjusted for age and gender, model 2 was additionally adjusted for dialysis vintage and comorbidity (history of diabetes mellitus and cardiovascular diseases), and model 3 was additionally adjusted for presence of RKF defined as daily urine volume > 200 mL/day. *P* for trend test was conducted by including the nutritional indexes quartiles as an ordinal score to the regression models. We evaluated the discriminative ability of each model using Harrell’s c-index^31^. Fourth, we divided the patients into four groups based on the median values of GNRI and Cr index and compared the risk estimates for all-cause and cardiovascular mortality among the groups as follows: patients with higher GNRI and higher Cr index; patients with higher GNRI and lower Cr index; patients with lower GNRI and higher Cr index; and patients with lower GNRI and lower Cr index. The median values for GNRI and Cr index were 95.2 and 20.8 mg/kg/day, respectively. Lastly, we explored the baseline characteristics associated with “malnutrition”. In the current study, we defined patients with “malnutrition” when baseline GNRI and Cr index values were equal to or less than the first quartile point, respectively. Candidate predictors of “malnutrition” were age, gender, dialysis vintage, diabetes mellitus, history of cardiovascular diseases, single-pool Kt/V, nPCR, serum albumin, serum Cr, use of VDRAs, and use of phosphate-binders based on clinical experience and theoretical considerations. We did not conduct formal sample size calculations and used all the available data to maximize statistical power. We performed all statistical analyses using STATA (version 14.2; Stata Corp, College Station, TX, USA) software.

## Supplementary information

Supplementary material.

## Data Availability

The data used for this study cannot be made publicly available, even as a minimal data set, because they were obtained from a third party (Arbor Research Collaborative for Health) and contain sensitive information on participants, including gender, age, and self-reported socioeconomic data. However, data requests can be sent to Arbor Research via their website (http://www.arborresearch.org/AboutUs/ContactUs.aspx).
