# Supplementary material for: Geriatric Nutritional Risk Index (GNRI) and Creatinine Index Equally Predict the Risk of Mortality in Hemodialysis Patients: J-DOPPS
Source: Sci Rep. 2020 Apr 1;10:5756. doi: 10.1038/s41598-020-62720-6 (PMC7113241; doi:10.1038/s41598-020-62720-6)
Supplement: Supplementary file 1 — Supplementary material. [file 41598_2020_62720_MOESM1_ESM.pdf]

Supplementary Information

Geriatric Nutritional Risk Index (GNRI) and Creatinine Index Equally Predict the Risk of Mortality in Hemodialysis Patients: J-DOPPS

Shunsuke Yamada MD, PhD [1*], Shungo Yamamoto, MD, DTM&H, DrPH [2,3], Shingo Fukuma MD, PhD [4], Toshiaki Nakano MD, PhD [1], Kazuhiko Tsuruya MD, PhD [5], Masaaki Inaba MD, PhD [6]

1 Department of Medicine and Clinical Science, Graduate School of Medical Sciences, Fukuoka, Japan

2 Department of Healthcare Epidemiology, School of Public Health in the Graduate School of Medicine, Kyoto University, Kyoto, Japan

3 Institute for Health Outcomes and Process Evaluation Research (iHope International), Kyoto, Japan

4 Human Health Sciences, Graduate School of Medicine, Kyoto University, Kyoto, Japan

5 Department of Nephrology, Nara Medical University, Nara, Japan

6 Department of Metabolism, Endocrinology and Molecular Medicine, Osaka City University Graduate School of Medicine, Osaka, Japan

Supplementary Table 1. Summary of the results of the comparison between two nutritional indexes

	**Lower GNRI**	**Lower Cr index**
**Association determined by Cox proportional hazard risk model**		
**Increased risk in all-cause death** (Tables 2 and 3)	significant	significant
**Increased risk in cardiovascular death** (Tables 2 and 3)	not significant	significant
**Association determined by restricted cubic spline curve**		
**Increased risk in all-cause death** (Figures 2 and S2)	significant	significant
**Increased risk in cardiovascular death** (Figures S3 and S4)	significant	significant
**Baseline characteristics associated with "malnutrition" defined by low GNRI or Cr index category** (Table 4)		
**Body mass index**	Increased odds ratio (significant)	Increased odds ratio (significant)
**Normalized protein catabolic rate**	Increased odds ratio (significant)	Increased odds ratio (significant)
**Serum albumin**	NA	Increased odds ratio (significant)
**Serum creatinine**	Increased odds ratio (significant)	NA
**Serum C-reactive protein**	Increased odds ratio (marginally significant)	Not significant

Abbreviations: Cr, creatinine; GNRI, geriatric nutritional risk index; NA, not applicable. A *P*-value less than 0.05 was considered statistically significant.

SUPPLEMENTARY FIGURES AND FIGURE LEGENDS

Figure S1

(A) (B)

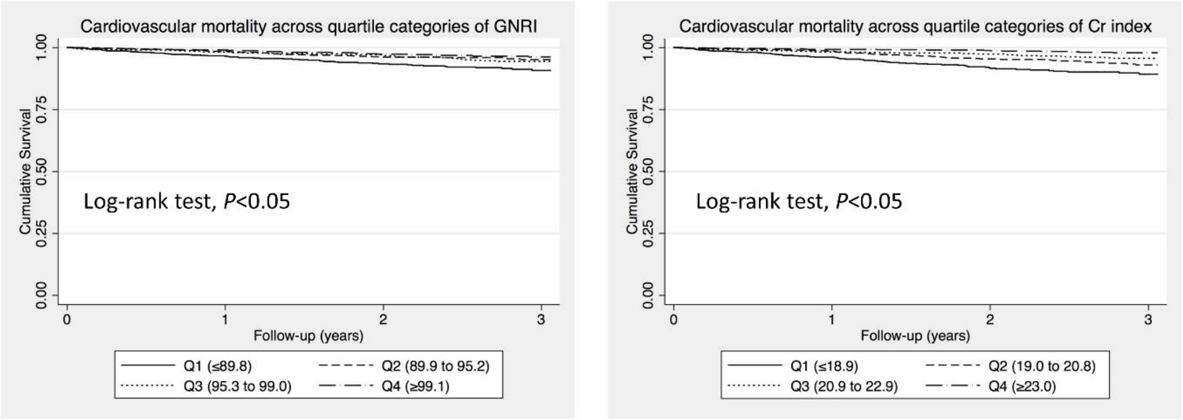

Figure S1. Kaplan-Meier curves for cardiovascular mortality stratified by the two nutritional indexes. (A) GNRI quartile and (B) Cr index. Log-rank test was used in the analysis. A two-tailed *P* value of <0.05 was considered to indicate statistical significance. Abbreviations: Cr, creatinine; GNRI, geriatric nutritional risk index.

Figure S2

(A) (B)

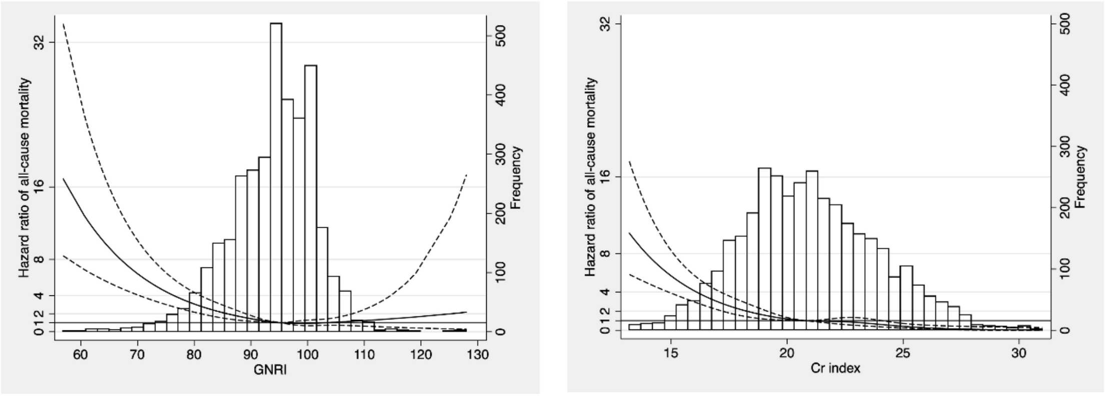

Figure S2. Multivariable-adjusted restricted cubic spline plots of HR for all-cause mortality according to the two nutritional indexes; adjustment for13 covariates. (A) GNRI and (B) Cr index. Solid lines represent HRs and dotted line represent 95% confidence intervals. The horizontal line corresponds to the normal reference HR of 1.0. Overall median values of the GNRI and Cr index were 95.2 and 20.8 mg/kg/day, respectively, and were chosen as the references. The multivariable-adjusted model was adjusted for age, gender, dialysis vintage, comorbidity (diabetes mellitus and cardiovascular diseases), systolic blood pressure, normalized protein catabolic rate, Kt/V for urea, blood hemoglobin level, and serum levels of C-reactive protein, calcium, phosphate, and parathyroid hormone. A two-tailed *P* value of <0.05 was considered to indicate statistical significance. Abbreviations: Cr, creatinine; GNRI, geriatric nutritional risk index; HR, hazard ratio.

Figure S3

(A) (B)

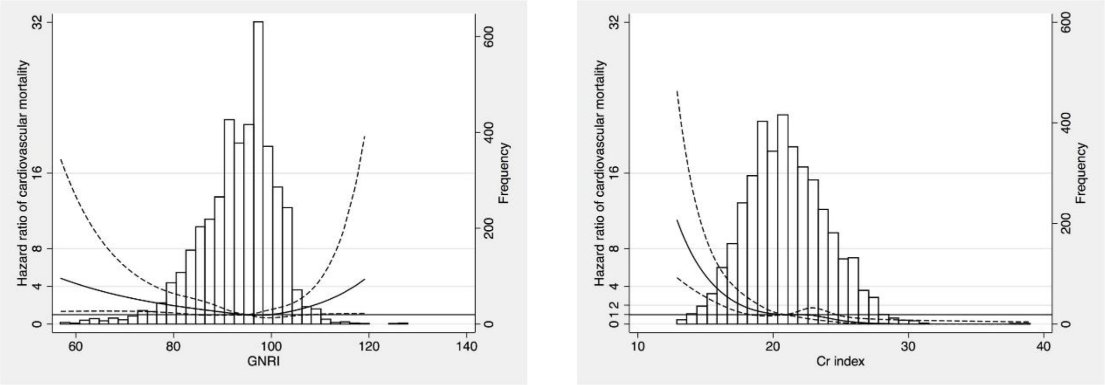

Figure S3. Multivariable-adjusted restricted cubic spline plots of HR for cardiovascular mortality according to the two nutritional indexes; adjustment for five covariates. (A) GNRI and (B) Cr index. Solid lines represent HRs and dotted line represent 95% confidence intervals. The horizontal line corresponds to the normal reference HR of 1.0. Overall median values of the GNRI and Cr index were 95.2 and 20.8 mg/kg/day, respectively, and were chosen as the references. The multivariable-adjusted model was adjusted for age, gender, dialysis vintage, and comorbidity (diabetes mellitus and cardiovascular diseases). A two-tailed *P* value of <0.05 was considered to indicate statistical significance. Abbreviations: Cr, creatinine; GNRI, geriatric nutritional risk index; HR, hazard ratio.

Figure S4

(A) (B)

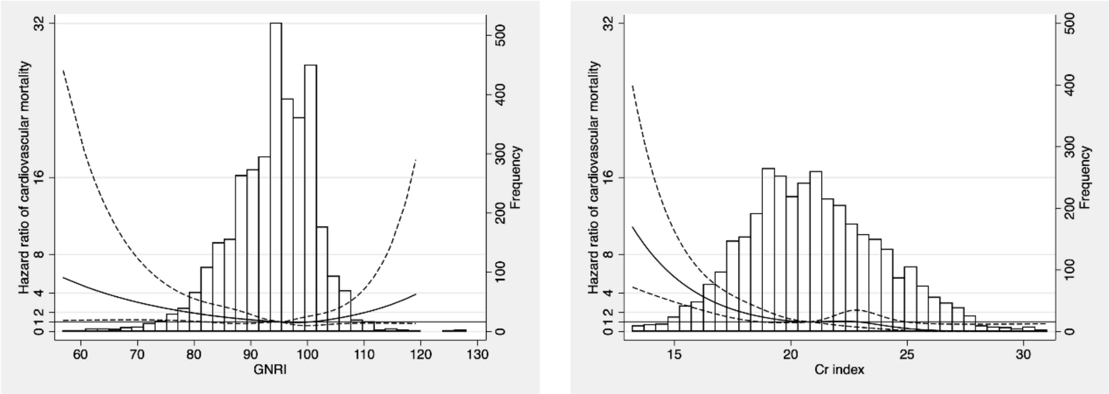

Figure S4. Multivariable-adjusted restricted cubic spline plots of HR for cardiovascular mortality according to the two nutritional indexes; adjustment for 13 covariates. (A) GNRI and (B) Cr index. Solid lines represent HRs and dotted line represent 95% confidence intervals. The horizontal line corresponds to the normal reference HR of 1.0. Overall median values of the GNRI and Cr index were 95.2 and 20.8 mg/kg/day, respectively, and were chosen as the references. The multivariable-adjusted model was adjusted for age, gender, dialysis vintage, comorbidity (diabetes mellitus and cardiovascular diseases), systolic blood pressure, normalized protein catabolic rate, Kt/V for urea, blood hemoglobin level, and serum levels of C-reactive protein, calcium, phosphate, and parathyroid hormone. A two-tailed *P* value of <0.05 was considered to

indicate statistical significance. Abbreviations: Cr, creatinine; GNRI, geriatric nutritional risk index; HR, hazard ratio.
